# Improving the Quality of Life of Patients With Medical Devices by a Timely Analysis of Adverse Events

**DOI:** 10.3389/fmed.2019.00056

**Published:** 2019-03-26

**Authors:** Urs P. Wyss

**Affiliations:** ^1^Department of Mechanical Engineering, University of Manitoba, Winnipeg, MB, Canada; ^2^Department of Mechanical Engineering, Queen's University, Kingston, ON, Canada

**Keywords:** medical devices, implants, adverse events, root cause analysis, joint replacements

## Abstract

Implanted and non-implanted medical devices, including artificial joints, are widely accepted to improve the quality of life of patients. While implant survival rates of over 80% can be accepted for artificial joints, there is still a large need to achieve higher survival rates at 15 years or longer to reduce the need for revisions due to implant failure before the end of the patient's life. Therefore, artificial joints are constantly improved with design changes and new designs, including modified or new materials. Most of these improvements perform as expected, but there are still cases where previously unknown failures occur, requiring premature revisions. A few examples of such unsuccessful improvements in the last 20 years are mentioned in this technical case report. The main focus of this paper is on an acetabular cup that was recalled due to unexpected revisions after a few weeks to a few months *in vivo*. The main reason for the revisions were small amounts of an oily residue containing endotoxins trapped inside the porous coating applied to the cup to facilitate bone ingrowth. The cup was recalled within 4 months after the company become aware of the problem, and prior to knowing exactly why the cups were failing early. The root cause analysis took several more months to complete. The lessons learned during the analysis are discussed so that similar events in other implantable medical devices can be avoided. The acetabular cup case aims to highlight that a timely root cause analysis, triggered by very few unexplained revisions, will benefit patients and improve the quality of life.

## Introduction

Implanted and non-implanted medical devices such as pacemakers, artificial joints, fracture fixation devices, dental implants, catheters, syringes, artificial limbs, braces, wheelchairs, *etc*., have become widely accepted to improve the quality of life for patients. These devices perform very well, depending on the application, for hours, days, months, or many years.

The focus of this paper is on artificial joints, where patient survival rates of 80% or higher after 15 years of use can be expected. An 80% implant survival rate means that up to 20% of today's implants must be replaced after <15 years of use, and implant revisions (replacement of the original, so-called primary implant) are often not as functional as primary implants. Therefore, a great need exists for survival rates much higher than 80% and for artificial joints that last twenty, 30 or more years, so that no revision is required before the end of the patient's life. While some revisions are necessary for reasons that are not related to the implant *per se*, those that are caused by the implant must be reduced.

Artificial joints have continuously been improved by more realistic modeling and simulation work, and improved *in vitro* testing. Pre-operative techniques are based on the understanding of the anatomy and physiology of normal and pathological structures that the artificial joints are replacing, and how these structures are being modified under the influence of the implant. Earlier analysis and understanding of adverse events has significantly contributed to the improvement of pre-operative evaluation methods. Often it would take months or even years, before adverse events leading to premature revisions were understood well enough to develop better modeling and simulation work, and more accurate *in vitro* testing protocols. The time it takes to fully understand adverse events means that tens of thousands of artificial joints that put patients at a higher risk for revision than necessary are still implanted.

Recent adverse events with artificial joints will be briefly described. The recall of the Inter-Op cup in December 2000 will be described in more detail. Legal issues were the main reason why not much was published about that event. The lessons learned from the Inter-Op cup event are still not well-known today, and the possibility of similar problems occurring with other artificial joints cannot be excluded.

## Recent Adverse Events

The DePuy ASR artificial metal-on-metal hip joint (Articular Surface Replacement) was sold since 2005 to more than 90,000 persons worldwide, and the device was voluntarily recalled by the company in August of 2010 (1, 2). There were reports of higher than normal revision rates for over 1 year before the recall. The reasons for the higher than normal revisions were not obvious, but it appeared that they were related to the metal-on-metal articulation of the device. The multi-billion dollar settlements with the patients after the recall were costly for the company, but the real cost was to the patients who required a premature revision with all the associated risks of an additional surgical intervention. An earlier recognition of the cause could have saved thousands of patients from suffering from a potentially faulty implant.

Another recent adverse event was the Zimmer Durom hip replacement where the cementless acetabular cup did not grow in properly in all cases, and eventually came loose, requiring revision surgery (3). There have also been reports of a higher ion release with the large diameter Durom hip (LDH). The company took the device from the market in 2012 and offered to settle outstanding lawsuits.

It has been known since at least 1992 (4) that corrosion can occur in modular hip implants, in particular between the implant head and the neck. The corrosion was noticed at revision surgery, but it was not believed to be a major concern. More corrosion in a larger number of revised implants was observed in larger heads that were introduced by several manufacturers since 2000. Higher corrosion at the head-neck taper connection appears to lead to an adverse tissue reaction due to the released corrosion substances. Several factors, such as material properties, surface structure, tolerances, the toggling moment, and others are responsible for the problems (5, 6). Much has been learned about head-neck corrosion in the meantime by a detailed retrieval analysis, simulation and modeling work, as well as *in vitro* tests. Nevertheless, there is still a need for better understanding why the corrosion occurs, and how it can be prevented, to eliminate corrosion at modular connections as a problem requiring revisions.

In addition to adverse events related to hip implants, other recent adverse issues also occurred with e.g., the Zimmer Persona trabecular metal tibia plate that was voluntarily recalled in 2015 (7). The reason was an increase in complaints of radiolucent lines and loosening. Another recent recall was the Zimmer Biomet Comprehensive Reverse Shoulder (8).

It is important to remember that the majority of artificial joints and other implants function well and remain in the body for a very long time. Frequent technical and/or manufacturing modifications and new implant designs aim to improve function and longevity. Some, however, do not work as expected and require early revision surgery, while others must be recalled and taken off the market immediately. Therefore, it is crucial to detect and understand adverse events as early as possible, so that as few as possible additional devices are implanted. Understanding adverse events in a timely manner will aid in the development of better modeling and simulation work and more realistic *in vitro* testing, ultimately improving *in vivo* success and patient outcomes.

## Inter-Op Recall on December 5, 2000

### The Inter-op cup

The Inter-Op cup was manufactured by Sulzer Orthopedics, Inc. in Austin, Texas. The Ti-alloy cup was hemispherical and coated with cancellous-structured Titanium (CSTi) on the bone side, allowing for bone ingrowth. The bone was reamed slightly smaller than the cup, so that a press-fit seating for primary fixation could be achieved during surgery. Screws could be used for additional initial primary fixation. The inside of the cup allowed for the insertion of a metal-on-metal or polyethylene articulation.

### Disclaimer

The description of the Inter-Op cup recall, how it happened and what was learned, is based solely on the knowledge of the author, who was Vice President of Research at Sulzer Orthopedics when it happened, and who coordinated the internal company investigation that followed the recall. The author feels strongly that the company dealt with this adverse event in a very timely manner, making the voluntary recall only a few months after it first became aware of a potentially higher failure rate of this particular cup. Of course, in hindsight, it is easy to argue that an even earlier recall would have saved many patients from receiving a faulty device.

### Investigations Before the Recall

**Figure 1:**
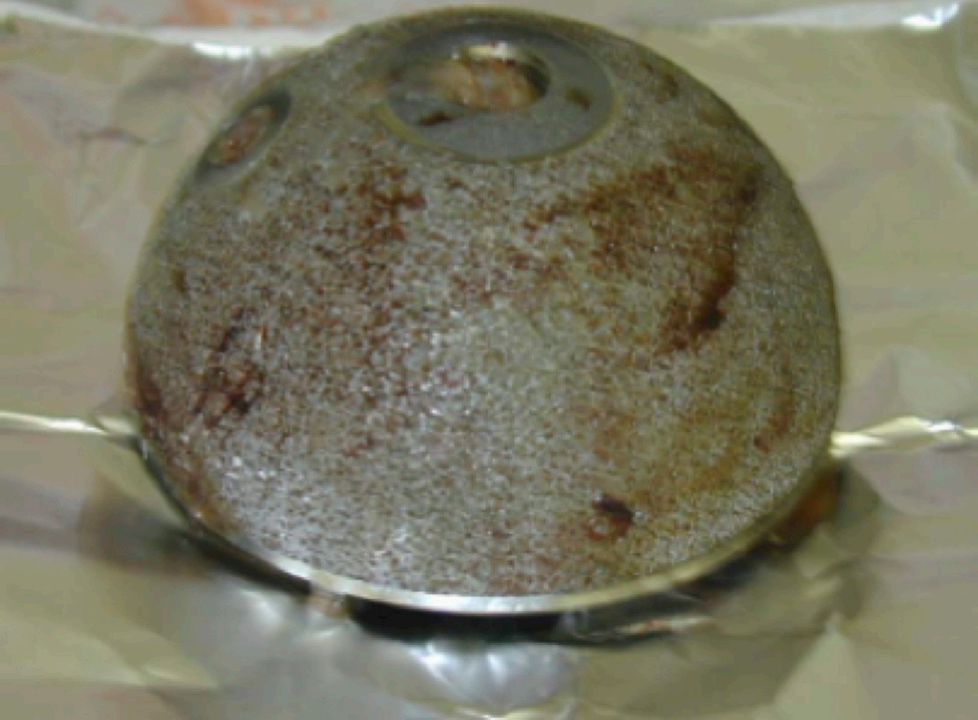
Revised Inter-Op cup after 11 months *in vivo* showing no bone ingrowth and a few areas with a red and gray gel-like residue.

In the summer of 2000, the product manager of the Inter-Op acetabular cup was informed by a surgeon from Los Angeles that a few unexpected early revisions had to be made in hip implants with a metal-on-metal articulation. The patients complained within weeks after surgery about pain. Some patients already showed reddening and swelling near the hip joint, which appeared to be a localized inflammatory reaction near the implant, and no positive bacterial culture could be detected. The surgeon was aware that in Europe between ten and twenty metal-on-metal articulation revisions were made, and that there was concern the metal-on-metal articulation was responsible. This was one reason why he contacted the company soon after he had to perform the first few unexpected revisions. As soon as the president of the company was made aware of higher than expected revisions, a task force consisting of project management, development, research, clinical studies, manufacturing, biology and legal was formed. The surgeon who made the company aware of the adverse events sent the retrieved cups to Dr. Pat Campbell, an international retrieval analysis specialist, for neutral analysis (Figure 1). Within a few weeks it was possible to rule out tolerance issues in the cup and/or the instruments as the possible cause of the problems, either on the articulation side and on the bone side. Adverse reactions due to the metal-on-metal articulation, first thought to be a possible cause, could be ruled out as a number of the same cups with a polyethylene articulation also had to be revised due to similar symptoms.

The time between the end of September and the recall on December 5, 2000 was very difficult, as the number of revisions started to increase and the company was at a loss about what was causing the problems. Recalling the Inter-Op cup, with all the consequences that a recall triggers, was discussed as the right thing to do, however, recalling an otherwise successful device without understanding the root cause of the problem would have been problematic. In November of 2000 extraction studies looking at residues on some off-the-shelf stored cups showed small amounts of oily residue. The oily residue of a few milligrams was trapped in the CSTi surface that has about 50% porosity. Dr. Campbell (retrieval analysis specialist) and Dr. Mirra (pathologist) from Los Angeles, who received many of the revised cups, suggested to the company that a biological problem was the most likely cause for the adverse reaction leading to pain and early revision. The absence of finding any other possible causes, and the fact that the revisions started to increase, led the company to make a voluntary recall of the Inter-Op cup on December 5, 2000. The reason for the recall was communicated as unacceptable levels of oily residues. Of the 25,000 cups that were recalled 17,000 were already implanted.

### Investigations After the Recall

#### Oily Residue in Porous CSTi Structure

The analysis of the residue was continued after the recall, on hundreds of not yet implanted cups in the company laboratory, and in two independent laboratories. The data was put into groups based on lot number and manufacturing and cleaning processes. The lot numbers of the revised devices were compared with the data from the off-the-shelf devices. This resulted in the following observations:

- Oily residue levels between a few mg and occasionally as high as 50 mg were found in manufacturing lots going back to 1997
- Most revisions were in one manufacturing group, where nitric acid passivation had been eliminated, as it was determined that nitric passivation did not enhance the existing self-passivation of Ti-alloy
- There were fewer revisions in cups manufactured within the first few weeks after the summer holiday shut down of manufacturing

**Figure 2:**
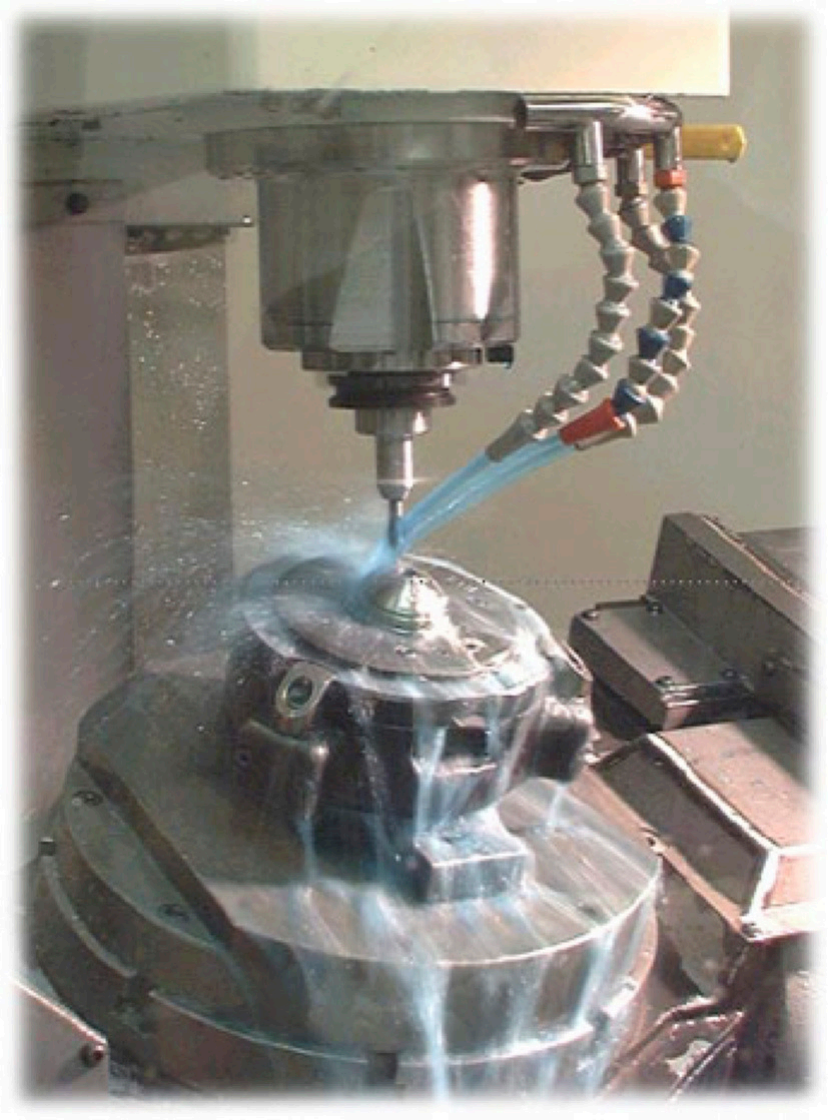
Coolant applied during machining finishing of the cup after porous coating.

The source of the oily residue was in the machine used to finish the parts after porous coating (Figure 2). The CSTi porous coating requires very high temperatures under vacuum to eliminate all non-metallic residues. Ideally, machining should not be necessary after porous coating in order to avoid contamination of the porous bone ingrowth structure. Oily residue can be cleaned from smooth surfaces during the final cleaning process, but it is not possible to completely clean a porous structure once it has been contaminated. The cooling fluid used during machining can contain small amounts of oil from the way bed of the machine, as well as the machine's gearbox and hydraulics. All manufacturing groups of the Inter-Op cup required some machining after porous coating, leaving various amounts of oily residue in the porous structure, even after the final cleaning process (9).

Very few revisions were observed in the groups where nitric acid passivation was applied after porous coating, as the nitric acid largely eliminated the oily residue within the porous structure. The holiday shut down was used to clean all machines and was also the time the cooling fluid was replaced. This meant that after the holiday break, the cooling fluid used contained very little oily residue. The different oily residues were identified and assessed for toxicity. Very small amounts of toxic additives could be detected, but in such small amounts that it could not explain the adverse tissue reaction observed in the retrieved devices. The pathologist Dr. Mirra from Los Angeles also did not feel that the oily residue alone could explain the inflammatory response requiring a revision of the cup. Endotoxins, sterile residues from bacteria, were already considered before the recall as a possible cause for the adverse tissue reaction. Endotoxin tests on finished off-the-shelf cups, however, were all negative.

## Endotoxins

Endotoxins are a toxin associated with the outer membranes of certain gram-negative bacteria. They are released upon disruption of intact bacteria (death, cell lysis). Their presence in the blood stream may cause septic reactions, and high concentrations can, amongst other reactions, lead to very serious intravascular coagulation or blood clotting (10). Therefore, standards exist for testing of cardiovascular medical devices, which dictate acceptable lower limits of endotoxins. Tests for endotoxins use the limulus amoebocyte lysate (LAL), which tests the fluid extract after immersing the medical device in the water, usually using ultrasonic cleaners. No standards or acceptable limits exist for endotoxin tests for orthopedic devices. Nevertheless, the Inter-Op cups were tested before and after the recall for endotoxins, but none could be detected.

An oily residue within the CSTi porous coating was given as the cause for the adverse tissue reactions in patients requiring cup revisions at the time of the recall. Only in the months after the recall was it possible to investigate the oily residue further, in order to understand the failure mechanism. It was already known by the assessment of Drs. Mirra and Campbell that the failure was likely be caused by an inflammatory process.

Coolant fluid was tested for endotoxins from a number of tool machines used for machining after porous coating. Various levels of live and dead bacteria (endotoxins) were found, depending on how much time had elapsed since the last coolant replacement. Several dozens of different bacteria were found when the air and many surfaces in the plant were tested. This explains how the bacteria were able to get into the coolant of the tool machines.

The coolant is a water-based, warm solution, *i.e*., an ideal environment for bacteria growth. These bacteria also mixed with the oil, leading to an endotoxin-loaded oily residue left in the porous coating of the cup. The subsequent washing was unable to remove all residues from the porous structure. The sterilization process took care of the live bacteria, but did nothing to the endotoxins. No endotoxin could be detected with the LAL test, as no oily residue could be extracted during the ultrasonic cleaning. Once the cup was implanted, it appears that when the body fluid came into contact with the endotoxin contaminated oily residue in the cup, it started an inflammatory reaction, preventing the bone from growing into the CSTi porous structure. This led to the loosening of the cup within a few weeks to a few months.

About a third of the 17,000 patients that had received an Inter-Op cup required a revision. Not all cups had the same amount of oily residue and endotoxins. This explains why fewer revisions were observed after the coolant replacement during the summer holiday shut down. Furthermore, some patients might have already had antibodies against some of the bacteria, leading to a lower or no inflammatory reaction. It was also learned during the investigation of the root cause that mineral oil acts as an adjuvant when mixed with endotoxin leading to a much stronger inflammatory response than endotoxin alone.

## Vitality of Bone After Revision

**Figure 3:**
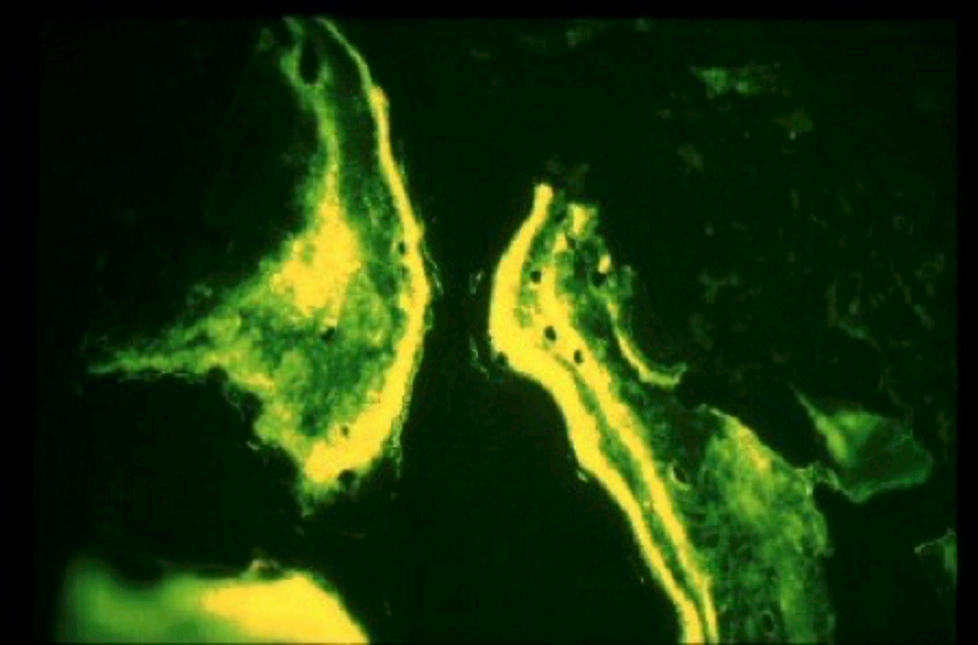
Tetracycline labeled bone chips showing the dual band of live bone.

The annual meeting of the American Academy of Orthopedic Surgeons took place, almost 3 months after the recall, in February of 2001. A few hundred revisions had already been made at that time, and the surgeons were wondering how deep into the bone the adverse tissue reaction had spread. Therefore, a tetracycline double labeling bone study was initiated in January of 2001 (11). Patients scheduled for revision surgery that had consented to the study were injected with tetracycline 2 weeks before surgery, and a second time again immediately before surgery. A thin layer of bone was removed after the cup had been taken out until fresh bleeding bone appeared. The bone chips were then analyzed for chips showing two bands, indicating that the bone was alive and growing (Figure 3). The results showed that only a thin layer of bone below the contaminated cup was compromised, and it was possible to ream it out without problems. This was very important for surgeons, who were faced with having to revise patients who developed pain and implant loosening due to the affected cup. The author, an engineer, was not aware that the revisions were abnormally difficult.

## Lessons Learned

The recall of the Inter-Op cup was an adverse event that took about 4 months from first reports about problems to the voluntary recall of the cup. While this was, in the opinion of the author, a timely process, one might question why the company did not act even earlier, preventing many patients from receiving a faulty device. Much was learned during the analysis of the root cause of the problem, but it could not be published for legal reasons. Here are a few of the lessons learned:

- It was important to recall the product once it appeared obvious that the Inter-Op cup was the reason for the majority of the premature revisions, even without fully understanding the failure mechanism
- It was crucial to send the retrieved cups to a specialized laboratory for independent analysis. This gave the surgeons confidence, as some did not trust the company to be completely open about the findings
- The adverse reactions endotoxins can cause when they get into porous structures of implantable medical devices is much better understood as a result of the implant failure and subsequent root cause analysis.
  **Recommendation:**
  - Any possibility of endotoxin contamination of rough or porous coating must be avoided, as it is extremely difficult to remove endotoxins from such surfaces. Endotoxins can enter porous surfaces during machining, through final cleaning in the case that the industrial water contains endotoxins, or through the air if the otherwise finished implants are not handled in a sterile environment before packaging
  - The industrial water upgraded from regular city water must be routinely tested for endotoxins
- Small changes in the manufacturing process should not be underestimated, and a thorough risk analysis is very important when any changes are made
- Relying on existing standards and tests is not sufficient, as with every change or potential improvement, previously unknown adverse events can occur. Standards and published *in vitro* tests are typically years behind the current knowledge, as it takes time to incorporate new knowledge into a standardized test.
- It is crucial to review the literature thoroughly about any aspect that could lead to an adverse event of a medical device. An article by Hollingsworth and Atkins published in (12) describes a synovial inflammatory response to bacterial endotoxin. The company was not aware of this article; knowledge of the article might have helped to understand the failure mechanism at an earlier time point.
- It is essential to take one or only a few adverse events with a medical device seriously and quickly analyze the cause of the problem. In many cases, there might not be a problem with the device, but missing a problem can put many patients unnecessarily at risk for a premature revision. If there appears to be a problem with the device, action should start without delay.

## Conclusions

It is important to remember that hundreds of thousands of patients experience a long lasting improvement in their quality of life after joint replacement. Nevertheless, there are routinely changes in existing and new devices to further improve the survival rate of artificial joints. Every change, however, bears the risk of unexpected consequences, which is why it is so important to perform excellent clinical studies, and to do a timely analysis of even the smallest number of adverse events. The Inter-Op example shows how important it was to quickly start the analysis of only a few initial unexpected revisions. The recall with all the severe consequences for the company, and more importantly the patients, was made only months after first hearing about problems with the cup. Of course it can always be argued that the recall should have been made earlier. The root cause analysis involved assessing a large number of retrieved and off-the-shelf devices. It showed the potential problems with endotoxins, especially on rough or porous surfaces. Once there endotoxins can hardly be detected or removed. Doing everything possible to prevent endotoxins from attaching themselves to such surfaces is crucial. It would be useful if companies would be required to publish what was learned in the root cause analysis of every recall, as other companies would be prevented from marketing devices with similar problems. Ultimately, patients would benefit from getting fewer faulty artificial joints.

## Data Availability

All datasets generated for this study are included in the manuscript and/or the supplementary files.

## Ethics Statement

The article is a commentary on an adverse event scenario and associated root cause analysis in real human patients, i.e., not as part of a clinical or other study. No identifying information is included in the manuscript.

## Author Contributions

The author confirms being the sole contributor of this work and has approved it for publication.

### Conflict of Interest Statement

UW was Vice President of Research at Sulzer Orthopedics during the recall and root cause analysis events described in the manuscript.
